# Recent advances in understanding apicomplexan parasites

**DOI:** 10.12688/f1000research.7924.1

**Published:** 2016-06-14

**Authors:** Frank Seeber, Svenja Steinfelder

**Affiliations:** 1 FG16: Mycotic and parasitic agents and mycobacteria, Robert Koch-Institute, Berlin, Germany; 2 Institute of Immunology, Center of Infection Medicine, Free University Berlin, Berlin, Germany

**Keywords:** Apicomplexa, parasites, Plasmodium, T. gondii, CRISPR/Cas9

## Abstract

Intracellular single-celled parasites belonging to the large phylum Apicomplexa are amongst the most prevalent and morbidity-causing pathogens worldwide. In this review, we highlight a few of the many recent advances in the field that helped to clarify some important aspects of their fascinating biology and interaction with their hosts. *Plasmodium falciparum* causes malaria, and thus the recent emergence of resistance against the currently used drug combinations based on artemisinin has been of major interest for the scientific community. It resulted in great advances in understanding the resistance mechanisms that can hopefully be translated into altered future drug regimens. Apicomplexa are also experts in host cell manipulation and immune evasion. *Toxoplasma gondii* and *Theileria* sp., besides *Plasmodium* sp., are species that secrete effector molecules into the host cell to reach this aim. The underlying molecular mechanisms for how these proteins are trafficked to the host cytosol ( *T. gondii *and *Plasmodium*) and how a secreted protein can immortalize the host cell ( *Theileria* sp.) have been illuminated recently. Moreover, how such secreted proteins affect the host innate immune responses against *T. gondii* and the liver stages of *Plasmodium* has also been unraveled at the genetic and molecular level, leading to unexpected insights.

Methodological advances in metabolomics and molecular biology have been instrumental to solving some fundamental puzzles of mitochondrial carbon metabolism in Apicomplexa. Also, for the first time, the generation of stably transfected *Cryptosporidium* parasites was achieved, which opens up a wide variety of experimental possibilities for this understudied, important apicomplexan pathogen.

## Introduction

**Figure 1.**
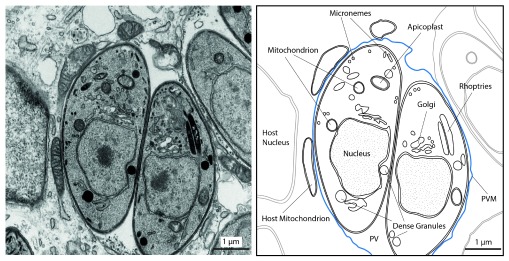
Morphology of intracellular *Toxoplasma gondii* tachyzoites. Transmission electron micrograph of the intracellular *Toxoplasma gondii* tachyzoite stage as one example of Apicomplexa. Shown are parasites inside an infected fibroblast host cell, residing within a parasitophorous vacuole (PV), which is surrounded by a membrane (parasitophorous vacuolar membrane, PVM, in blue) (left). On the right, some host and parasite organelles are outlined. The apicoplast is a plastid-derived organelle harboring some essential metabolic pathways. Micronemes are specialized secretory organelles at the apical end that store proteins important for gliding motility and host cell invasion ^130^. The role of dense granules and rhoptries are detailed in the text. This basic organellar setup is shared with *Plasmodium* sp.

Apicomplexa comprise a large phylum of single-celled, obligate intracellular protozoan organisms that all have a parasitic lifestyle. Among the more than 6000 named and probably more than one million unnamed species ^1^ are some of great public health and economic relevance, since they cause severe diseases in humans and livestock, affecting millions each year ^2– 6^. Therefore, increased knowledge about their biology in general, e.g. to exploit vulnerabilities, and their interaction with the host organism, e.g. to stimulate the immune system, is of great importance and promises major benefits in understanding and combating the diseases they cause. In addition, they possess a fascinating biology as intracellular eukaryotes thriving within another eukaryotic cell ( Figure 1), which clearly sets them apart from other pathogens like bacteria and viruses. Taken together, these are all good reasons to pay attention to these fascinating organisms. This review will focus on four important genera.

*Plasmodium falciparum* and four other *Plasmodium* species that affect humans cause malaria, a mosquito-transmitted, potentially deadly disease. According to the latest data from the World Health Organization (WHO), the number of deaths due to malaria has declined by 48% between 2000 and 2015 but the disease still causes the loss of ca. 438,000 lives each year of the 218 million people infected, mostly children under 5 years of age ^5^.

An estimated one-third of the human population is chronically infected with *Toxoplasma gondii* leading to toxoplasmosis. It can cause severe symptoms in newborns (e.g. encephalitis and ocular disease) when a previously non-infected mother contracts the infection during pregnancy by ingesting infectious stages of *T. gondii* via contaminated food, water, or dust ^7^. The latest numbers from WHO rank toxoplasmosis highest with respect to the overall lifelong disease burden among those foodborne diseases caused by protozoan parasites ^8^.

The same study identified cryptosporidiosis (caused by two *Cryptosporidium* species, *Cryptosporidium hominis* and *Cryptosporidium parvum*) as the second most important disease in this class. Worldwide, each of these species infects 8–10 million people per year, but in addition they also cause considerable disease in livestock.

Lastly, *Theileria parva*, a tick-transmitted apicomplexan of ruminants, leads to the death of more than 1 million cattle per year in sub-Saharan Africa, causing costs of >300 million US$. This has severe socio-economic consequences for those regions ^4^.

*T. gondii*, and to a lesser extent *Plasmodium* sp., has also gained increased attention from cell biologists owing to a number of unique mechanisms of host cell entry, division, motility, etc., thereby becoming model organisms for such aspects ^9^. Both medical importance and model character are reflected by the overall citations in PubMed (several thousands from January 2013–December 2015 for the four taxa), making it impossible to cover even the most interesting discoveries of the last 2–3 years in all areas of apicomplexan research in reasonable depth. Consequently, only a few, admittedly subjective highlights in the fields of cell and molecular biology, biochemistry, and immunology as well as aspects concerning drugs will be mentioned here. These were selected because they are expected to make a major impact in the following years or offer explanations for puzzling unexplained observations in apicomplexan biology.

## Methodological advances as game-changers

Many new findings require new technologies at first, and with the advent of next-generation sequencing (NGS) techniques ^10^, the number and quality of apicomplexan genome sequences and respective transcriptomes have been growing considerably, allowing insights and discoveries that would have been hard or impossible to gain a few years ago ^11– 13^. The genetic tools are most advanced for *T. gondii* and *Plasmodium*, and further specifics can be found in recent articles ^14– 17^. Moreover, the Clustered Regularly Interspaced Short Palindromic Repeats (CRISPR)/CRISPR-associated protein 9 (Cas9) genome editing system ^18^ and related techniques started a wave in 2012 ^19, 20^ that swept over the life sciences and since then has also reached the Apicomplexa. It has allowed targeted deletions, mutations, or gene additions so far in *Plasmodium, T. gondii*, and *Cryptosporidium* with unprecedented ease ^21– 25^, as will be illustrated here for the latter.

*Cryptosporidium* sp. have some unique biological aspects within the phylum, which are of great evolutionary interest, like the presence of only a rudimentary mitochondrion (mitosome) or the absence of the secondary plastid called apicoplast ^26, 27^. However, the lack of protocols for efficient long-term propagation in cell culture, reverse genetics, and thus methods to mark the parasites with fluorescent proteins has long precluded a broader analysis of many of those aspects. Application of CRISPR/Cas9, together with a number of smart optimizations of established techniques, plus the transfer of transfected parasites directly into the intestine of immune-deficient mice and their subsequent selection therein, changed the game ^22^. This allowed for the first time the establishment of stably transfected *C. parvum* parasites and opens up a whole variety of options. For example, parasites expressing a reporter enzyme like luciferase will enable comprehensive drug screening efforts, allowing the tackling of an urgent need. Likewise, generation of genetically attenuated parasites by multi-gene deletions can be envisaged as a means to develop oral vaccines for livestock (or even humans). Effective vaccines for the former would minimize the contamination of the environment with excreted infectious oocysts. Finally, many fascinating cell biological aspects can now be followed and analyzed using fluorescent sporozoites.

## All about artemisinin in a nutshell: biotechnological production, mode of action, and the emergence and nature of *Plasmodium* drug resistance

In 2015, the Nobel Prize in Physiology or Medicine was in part awarded to Chinese scientist Youyou Tu for her major contributions to the discovery of artemisinin in the 1970s ^28, 29^. It is the ingredient of a traditional Chinese herbal anti-malarial treatment that, when metabolized *in situ* to dihydroartemisinin, very efficiently kills *Plasmodium* sp.. Artemisinin combination therapies (ACTs) are currently the drugs of choice and recommended by WHO for treating *P. falciparum* infections worldwide, largely because drug resistance against other available compounds precludes their further use in many areas of Africa and South-East Asia (SEA) ^30^. ACTs consist of fast-acting artemisinin (or its derivatives) and less-potent, long-acting partner drugs. Artemisinin’s mode of action is unique in the sense that not a single protein or cell component but a multitude of parasite molecules are targeted by the compound via the generation of highly reactive endoperoxide-derived radicals ^31^. These affect at least 124 parasites but few, if any, host proteins (at the level of currently applied experimental resolution), as a recent study reported ^32^. This probably leads to the observed cellular stress response and increased molecular tagging of the affected proteins for disposal by the “cellular garbage can”, the proteasome ^33^. Many of these proteins are known or suspected to be essential for parasite growth, and their concerted damage by an unselective mechanism like oxidative damage could be expected to make it fairly difficult for the parasite to develop resistance (see below).

Only one plant is known to produce artemisinin, *Artemisia annua* L., and extraction yields do not exceed 0.5%. Therefore, the molecular deciphering of its biosynthesis ^34^ and the subsequent biotechnological production of artemisinic acid, the key precursor from which artemisinin and other derivatives can be derived by straightforward chemical synthesis, was a major breakthrough ^35, 36^. The genes required for this pathway were engineered into a yeast strain that can now produce artemisinic acid with a yield of 25 g/L of fermentation broth ^36^. Notably, although developed by a company, all intellectual property rights have been provided free of charge.

Unfortunately, this success story has recently been dampened by the emergence (again!) of resistance phenotypes in SEA ^37, 38^. Resistance is defined as a parasite clearance half-life of at least 5 hours following ACT treatment, whereas non-resistant *Plasmodium* parasites are all killed earlier. The problem lies in the fact that delayed clearance (parasites are still killed, but slower) exposes the surviving organisms to the second drug in ACT, increasing the chance that resistance to this partner compound develops. Clearly, understanding the mechanism of artemisinin resistance is of utmost importance to be able to counteract and change drug regimens or composition.

**Figure 2.**
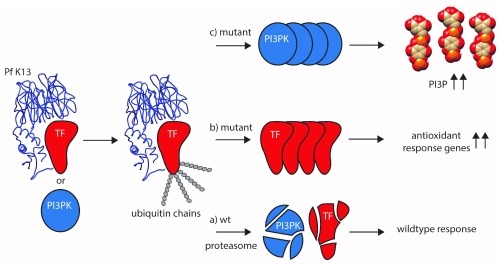
Proposed artemisinin-resistance mechanisms. In wild-type (wt) parasites, binding of K13 (blue, based on 10.2210/pdb4yy8/pdb) to an as-yet-unknown transcription factor (TF, red) usually leads to the tagging of TF with ubiquitin, followed by its subsequent degradation via the proteasome (a). This regulatory mechanism is abolished when mutated K13 is no longer able to bind efficiently to TF, thereby preventing ubiquitin tagging and subsequent proteasomal degradation (b). This results in increased transcription of genes and production of the corresponding proteins involved in antioxidant defense. Their activity allows counteracting the oxidative damage brought upon them by artemisinin. In an alternative model ^43^, the low phosphatidylinositol-3-phosphate (PI3P) levels usually found in artemisinin-sensitive parasites is maintained by K13 binding to its kinase phosphatidylinositol-3-kinase (PI3PK, blue), its ubiquitination (similar to TF) and subsequent degradation (a). Loss of this regulation leads to increased PI3PK levels, followed by a buildup of PI3P (c). Higher levels are presumably responsible for parasite growth in the schizont stage via promoting membrane biogenesis and fusion events during parasite growth. In addition, artemisinin combination therapy (ACT) is able to directly inhibit PI3PK. Both mechanisms could also benefit from the observed slowed-down growth and upregulation of the unfolded protein response pathway ^44^, giving treated parasites more time to repair damaged proteins before they progress through the rest of their lifecycle in red blood cells. Figure adapted from 37.

Here, NGS and related techniques come into play. Sequencing resistant *P. falciparum* isolates directly from patients, a number of recent studies provided solid evidence for multiple mutations in a gene called *kelch13* ( *K13*), which are associated with increased resistance ^39– 42^. Transcriptomics identified an additional protein presumably involved, phosphatidylinositol-3 kinase (PI3K) ^43^ (for details, see Figure 2).

Another transcriptomics study of 1000 (!) clinical *P. falciparum* isolates provided evidence that a population of parasites exists that is slowed down in growth and shows an upregulated so-called “unfolded protein response” pathway ^44^. This might allow parasites to repair proteins that were oxidatively damaged by artemisinin’s mode of action (see above) and progress through the rest of their lifecycle in red blood cells. The reduced growth would give them more time to do so. Puzzlingly, this report found no association between PI3K transcript levels and either parasite clearance half-life or *K13* mutations. Evidently, more efforts are required to reconcile all the observations and to fully understand resistance development. Nevertheless, in a very short time, from the initial observation of delayed clearance times of ACT in SEA to the current studies, immense progress has been made in understanding the obviously very complex artemisinin resistance mechanism(s). Technological breakthroughs were just there at the right time to face this challenge.

## Mitochondrial metabolism – similar but still significantly different

Condensing parasitism to the meaning of the Greek word παράσιτος (parásitos; person who eats at someone else's table), and regarding biochemistry as the underlying science to reveal this eating behavior, there is an obvious and long-standing interest in understanding apicomplexan metabolism, not least because enzymes can make good drug targets ^45^. Again, technological advances in the field of metabolomics, together with gene knockouts, have greatly helped finding some answers to who eats what, when, and how.

Mitochondrial carbon metabolism in Apicomplexa is central to the generation of energy and several precursors of other pathways that occur outside the organelle, like pyrimidine and heme biosynthesis ^46– 49^. One fundamental energy-generating system in most eukaryotes is the tricarboxylic acid (TCA) cycle in this organelle. It leads to complete oxidation of carbohydrates, lipids, and amino acids, thus allowing much greater ATP generation through the electron transport chain (ETC) than e.g. breakdown of glucose via glycolysis. *T. gondii* and *Plasmodium* sp. can get along with an energy supply derived only from glycolysis as long as they are living in glucose-rich environments. In fact, most (but not all) TCA cycle enzymes in *P. falciparum* could be knocked out with only little influence on the blood stage forms ^50^. Nevertheless, for a number of reasons, it seemed that a TCA cycle and ETC were operating in both organisms. For instance, several recent studies ^50– 55^ have shown that the development of *Plasmodium berghei* (a rodent model for *P. falciparum*) in the mosquito is not possible without a functional TCA cycle or ETC (summarized in 47). These studies have highlighted the great flexibility of both *Plasmodium* sp. and *T. gondii* with regard to substrate utilization and adaptation of carbon metabolism to different host environments.

**Figure 3.**
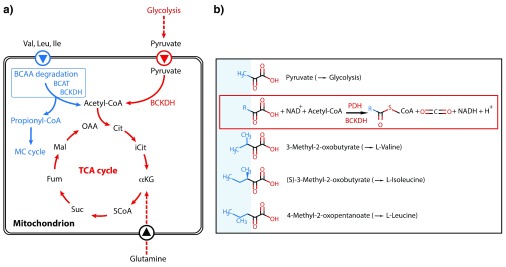
Feeding of the mitochondrial carbon metabolism. a) Current model of how the tricarboxylic acid (TCA) cycle is fed with substrates in both *Toxoplasma gondii* (blue) and *Plasmodium berghei* (red; in common with *T. gondii*) and converted to acetyl-CoA. The latter then enters the TCA cycle. BCAA degradation in *T. gondii* can lead to toxic propionyl-CoA, which could be detoxified by the 2-methylcitrate (MC) cycle. Its physiological importance for the different *T. gondii* stages is currently ill defined, as both BCAA degradation and MC cycle are dispensable in tachyzoites ^131^. Abbreviations: α-KG, α-ketoglutarate; BCAT, branched-chain amino acid transferase; BCKDH, branched-chain keto acid dehydrogenase; Fum, fumarate; Cit, citrate; iCit, isocitrate; Mal, malate; OAA, oxaloacetic acid; PEP, phosphoenolpyruvate; Suc, succinate. Figure adapted and redrawn from 51. b) Structural similarities of substrates for pyruvate dehydrogenase (PDH) and branched-chain keto acid dehydrogenase (BCKDH), respectively. The framed reaction scheme illustrates the overall generation of CoA compounds and CO _2_ from the substrates catalyzed by the two enzymes.

However, an unresolved mystery was the experimentally well-proven absence of a key enzyme complex, pyruvate dehydrogenase (PDH), in the mitochondrion of Apicomplexa that would allow these organisms to feed glucose-derived pyruvate into the TCA cycle ^47, 48^ ( Figure 3a). This longstanding conundrum has now been solved, and it could be shown that a structurally related mitochondrial enzyme complex, branched-chain alpha-ketoacid dehydrogenase (BCKDH) usually involved in the degradation of branched-chain amino acids (BCAAs), has evolved to also take over the PDH task, generating acetyl-CoA from pyruvate ^51^ ( Figure 3a). Since pyruvate and the usual substrates for BCKDH are structurally quite similar ( Figure 3b), it is likely that only few (so far unknown) mutations were necessary to acquire the required substrate specificity. It is an illuminating example that holes in metabolic pathways predicted from genome data are not necessarily an annotation problem but can reflect evolutionary processes of reductive evolution ^46, 48^.

Reductive evolution aims to reduce the considerable metabolic burden of gene and protein synthesis when the function of lost genes can be fulfilled otherwise ^56, 57^. Importantly, in this case, parasitism wasn’t the driving force, since free-living dinoflagellates that share a common ancestor with Apicomplexa are also devoid of a mitochondrial PDH but possess BCKDH ^46^. Interestingly, while *Plasmodium* sp. have lost the entire BCAA degradation pathway of enzymes operating before and after BCKDH ^46, 48^, thus saving even more on gene and protein synthesis, *T. gondii* as well as non-parasitic photosynthetic algae related to Apicomplexa have kept it ^58^.

## Understanding apicomplexan host cell manipulation – it all depends on protein export

One of the most fascinating aspects of the biology of Apicomplexa in general is their remarkable capacity to manipulate their respective host cells to suit their own demands. This causes changes in host cell signaling, nutrient delivery to the parasite, and evasion of host immunity ^59– 62^. Amongst the many strategies leading to this outcome, unique examples are provided by *T. parva* and *Theileria annulata*.

Both species have the ability to immortalize (transform) their host cells, which resembles in many aspects cancerous cell transformation. *T. parva* transforms bovine B and T lymphocytes, whereas *T. annulata*, besides B cells, also transforms macrophages and dendritic cells ^63^. Activation of the c-Jun pathway, a signaling cascade involved in controlling many cellular processes including proliferation ^64^, has been shown previously to be required for *Theileria*-induced transformation ^65, 66^. However, how this is accomplished at the molecular level has long been elusive.

Now, Marsolier *et al*. ^67^ have reported the identification of a parasite-encoded enzyme called peptidyl-prolyl isomerase (PPIase), a homologue of human parvulin (hPIN1), as the transforming agent. Upon export into the host cell, the *Theileria* PPIase, like the human homolog, binds to a host protein (an ubiquitin ligase that controls the levels of c-Jun), leading to its destabilization and subsequent degradation. This in turn results in higher levels of c-Jun and eventually leads to cell transformation. These surprising findings go beyond parasite biology, as they also define prolyl-isomerization as a conserved mechanism that is important in cancer development.

*T. gondii,* amongst other Apicomplexa, is also a known expert in manipulation of the host cell at various levels via the secretion upon host cell entry of effector proteins ^68, 69^, the number of which (currently around 80 ^58^) is still increasing ^70– 72^. The so-called rhoptry proteins (ROPs) are injected into the host cell cytosol during the actual invasion process via special organelles called rhoptries ^73^. A number of so-called GRA proteins derive from other organelles, the dense granules, and are delivered into the host cytosol after the parasite has formed its vacuole wherein it resides ^74^. Consequently, and in contrast to the ROPs, their mechanism of trafficking must include a way to pass this membrane structure. This aspect recently gained increased attention ^75^.

Precedence for this transport pathway came from the one that had been described for erythrocytic stages of *Plasmodium.* There, one molecular entity in the vacuolar membrane named “Plasmodium translocon of exported proteins” (PTEX) was recently described, which transports many parasite proteins into the cytosol of the red blood cell ^76– 79^. Delivery to PTEX depends in turn on prior export of proteins out of the parasite cell into the vacuolar space, which requires cleavage by a specific protease (plasmepsin 5) at a particular sequence motif called “Plasmodium export element” (PEXEL) ^79^. This PEXEL motif allows the identification of many but not all proteins that are to be exported into the host cytosol ^80^.

It made sense to also look for such a motif in *T. gondii* – and it was found for numerous known GRA proteins ^75^. Now, several groups recently reported the importance of export of *T. gondii* aspartyl protease ASP5 (the homolog to plasmepsin 5 in *Plasmodium*), which resides in the parasite’s Golgi and processes those GRA proteins containing the PEXEL-like motif ^70, 72, 81^. However, not all GRAs that depend on ASP5 for export contain a PEXEL-like motif. In *Plasmodium,* plasmepsin 5 is an essential gene already under *in vitro* growth conditions. This fact allowed the development of a very potent inhibitor of this crucial enzyme and that was subsequently used for structural studies ^82^. In contrast, *T. gondii* parasites with a deleted ASP5 grow fine in cell culture but are much less virulent in a mouse model than the highly virulent parental strain. The very same phenotype has been described for a knockout strain of the ASP5-processed GRA14 ^83^. Together, this indicates the crucial importance of a repertoire of exported proteins during a natural infection. One reason for this might be the observed reduced migration of infected dendritic cells (which are known to be misused by *T. gondii* as Trojan horses to reach the brain ^84^), thereby lowering the dissemination within the host ^72^. Interestingly, this disparate phenotype upon gene deletion of *in vitro* and *in vivo* growth, together with a severe impact on protein export into the host, is shared with another recently reported *T. gondii* parasitophorous vacuolar membrane (PVM)-resident protein, Myc regulation 1 (MYR1) ^85^. Whether MYR1 is part of the *T. gondii* “translocon of exported proteins” needs to be determined, since e.g. *Plasmodium* sp. lack a homolog of MYR1. In addition, a *T. gondii* homolog of the putative PTEX pore protein EXP2 ^86^, GRA17, is involved in the translocation of only small molecules through the PVM but apparently not of proteins ^87^. This indicates that both species use very different molecular complexes for protein export into the host cell.

Another distinctive difference to *Plasmodium* sp. is that in the absence of ASP5, certain GRA proteins fail to be exported into the host’s cytosol and then further on into its nucleus. Here, they cause large disturbances in the transcriptome of the infected cell ^70, 72^. *Plasmodium* sporozoites, the stage that infects hepatocytes, apparently do not possess dense granules ^61^. Nevertheless, large transcriptomic changes in liver cells occur upon infection ^88^. Apparently, in both parasite species different effectors accomplish similar things, i.e. they modify their host cells to optimize their own survival therein.

The *T. gondii* studies further indicate that many more exported effector proteins besides the known ROPs and GRAs need to be identified to fully understand the ways this parasite manipulates its host cell. The ASP5 mutants will be a valuable source in this respect. More studies will be required to understand the details and evolution of this crucial mechanism in the Apicomplexa ^89^.

## Innate immune defense always starts at the host-parasite interface

Innate immune responses are the immediate answer to an infection. They are triggered by the recognition of pathogen-derived molecules via evolutionarily conserved host receptors ^90^. In the last two decades, numerous studies found that innate sensing of apicomplexan infection is mediated by membrane-bound and cytoplasmic so-called pathogen recognition receptors (PRRs) such as Toll-like receptors (TLRs) and Nod-like receptors (NLRs) ^91, 92^. Early sensing of infection by these pathways in antigen-presenting cells bridges the innate and adaptive immune responses by licensing them to interact with naïve, antigen-specific CD4 ^+^ and CD8 ^+^ T cells to stimulate them to become effector cells capable of producing cytokines and/or cytotoxic molecules. Efficient control of *T. gondii* and *Plasmodium* sp. infections is mediated by T and NK cell-derived interferon-gamma (IFN-γ) ^93, 94^. This cytokine triggers the activation of very diverse effector pathways, e.g. NO production in infected host cells ^95^ or induction of immunity-related GTPase (IRG) proteins (a family of rodent IFN-γ-induced GTPases) or GBP proteins (IFNγ-inducible guanylate-binding proteins) that damage the PVM ( Figure 1), thereby mediating parasite death ^96– 101^. Recent studies emphasized the importance of GBP1 ^100^, GBP2 ^101^, and the cooperation of IRGs with GBPs ^99^ in cell-autonomous immunity and anti-parasitic resistance.

While the effects of IRGs on *T. gondii* in infected mice were known for some time, it became only recently apparent that laboratory mice and wild *Mus musculus* show enormous sequence diversity in particular genes of this family, with great and unexpected consequences for infection ^102^. For decades, *T. gondii* strains were defined as being highly virulent in various strains of common laboratory mice because even a single parasite could kill them within days. Unexpectedly, those strains totally failed to reproduce this phenotype in wild mice. This is largely due to a highly polymorphic IRG allele that confers resistance against virulent parasites by interfering with their virulence factors of the ROP family of protein kinases ^102^. The study is a striking example that lab mice and their wild counterparts can show very different responses to identical immunological challenges.

Protective immunity against *T. gondii* is governed by an IL-12-triggered Th1-type immune response, which involves NK cells, CD4 ^+^ effector cells, and CD8 ^+^ cytotoxic T cells as sources of IFN-γ ^93^. In order to induce its production by these cells, the innate arm of the immune system needs to sense the infection and relay this information into IL-12 production as an igniting factor for the ensuing Th1 response. Control of the infection is dependent on MYD88 ^103^, an adaptor molecule common to several TLRs, and the parasite-derived TLR-ligand *T. gondii* profilin ( *Tg*PRF). The latter binds TLR11, which triggers a signaling cascade that stimulates IL-12 production by dendritic cells ^59, 104^. However, TLR11 deficiency only modestly affects the survival of *T. gondii*-infected mice. Only recently was TLR12 shown to be involved in host resistance to *T. gondii* by recognizing *Tg*PRF ^105^ and cooperating with TLR11 to induce IL-12 in macrophages and dendritic cells ^106^.

**Figure 4.**
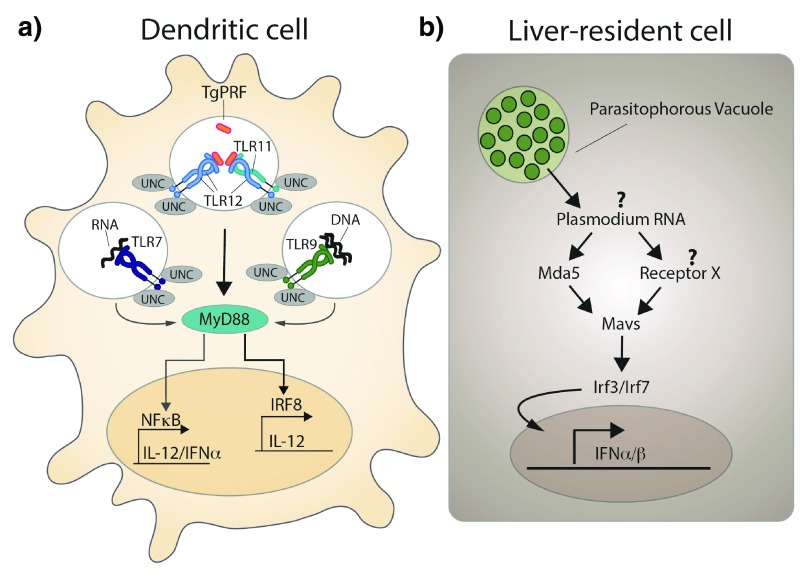
Innate sensing of *Toxoplasma gondii* and *Plasmodium* sp. by host cells. a) Simplified model of how dendritic cells sense *T. gondii* infection. Toll-like receptor 11 (TLR11) and TLR12 are the major receptors for the *T. gondii*-derived protein profilin ( *Tg*PFR). Interferon regulatory factor 8 (IRF8) ^+^ dendritic cells (DCs), in particular CD8 ^+^ DCs, have a crucial role in detecting *T. gondii* profilin and in the subsequent induction of interleukin (IL)-12 production downstream of myeloid differentiation primary-response protein 88 (MYD88). A novel MYD88-dependent signaling pathway that depends on activation of IRF8 is an explanation for the potent induction of IL-12 expression by CD8α ^+^ DCs that have been exposed to *T. gondii*, but the direct connection between MYD88 and IRF8 has not been established. In addition, both TLR7 and TLR9 have been implicated in detecting *T. gondii* RNA and genomic DNA, respectively. UNC93B1 (UNC), an endoplasmic reticulum-resident protein, has a central role in the function of all of the depicted endosomal TLRs and is essential for resistance to *T. gondii* (modified and redrawn from 132). b) Schematic representation of the proposed sequence of events occurring in liver-resident cells upon *Plasmodium* sp. infection. *Plasmodium* RNA triggers a type I interferon response via activation of the cytoplasmic RNA sensor Mda5 and other unknown receptors, mitochondrial antiviral signaling protein (Mavs), and the transcription factors interferon regulatory factor 3 (Irf3) and Irf7 (modified and redrawn from 111).

Other studies demonstrate the involvement of additional pathogen-derived molecules and PRRs in starting an immune response by contributing to the stimulation of IL-12 production. A recent study employed mice carrying a mutation in UNC93B1, a molecule involved in subcellular trafficking of endosomal TLRs ^107^. These mice are highly susceptible to experimental toxoplasmosis and their phenotype is not recapitulated by mice deficient in nucleic acid sensing (TLR3-/TLR7-/TLR9-deficient) but rather by mice deficient in TLR7/TLR9 and TLR11, highlighting the redundancy of pathogen recognition in those animals ^108^ ( Figure 4a). Humans lack TLR12 and harbor TLR11 as a pseudogene but produce high levels of pro-inflammatory cytokines in response to live phagocytosed parasites and parasite RNA and DNA. Thus, nucleic-acid-sensing TLRs seem to be the PRR operating in both hosts ^108, 109^, while recognition of *Tg*PRF by the TLR11/12 pathway is most likely an adaptation of rodents being a major intermediate host for *T. gondii* ^109^.

In contrast to *T. gondii*, which can infect virtually any nucleated cell, *Plasmodium* sp. are restricted to infecting hepatocytes and erythrocytes. Upon injection of *Plasmodium* sporozoites by the mosquito vector into the skin of the host, they first migrate to the liver and invade hepatocytes where they massively replicate. Early work on innate sensing of *Plasmodium* revealed that hemozoin-containing parasite DNA is recognized by TLR9 and mediates proinflammatory cytokine production by dendritic cells ^110^, but little was known about the hepatic immune response. Strikingly, and in stark contrast to the erythrocytic cycle, which causes the well-known malaria symptoms like recurrent fever, this initial contact between the parasite and its host cell is without clinical signs. This could be taken as evidence for an immunologically silent hepatic stage of the infection. However, recently it was shown that hepatocytes do respond to *P. berghei* sporozoite invasion with induction of a type I IFN response. Parasitemia in liver and red blood cells was increased and leucocyte recruitment was decreased in type I IFN receptor-deficient ( *Ifnar* ^-/-^) mice, highlighting the important role of type I IFN at this early phase of infection ^111^. The authors proposed a mechanism involving sensing of *Plasmodium* RNA by the cytoplasmic RNA receptor Mda5 and signaling via the mitochondrial antiviral signaling protein (Mavs) and the transcription factors Irf3 and Irf7, finally leading to transcription of IFN-α and IFN-β ( Figure 4b). A potential link among IFN-α, cell recruitment, and parasite elimination could be IFN-γ-secreting NK T cells, suggested by a recent study using the related species *Plasmodium yoelii* ^112^. However, a successful immune response often comes at a price, since a type I IFN response also causes malaria-associated immunopathology, such as liver damage and brain pathology ^113, 114^.

## Outlook

Obviously, we could give only a subjective glimpse of the recent exciting developments in understanding apicomplexan biology. What will come next?

One can probably barely overestimate the impact that CRISPR/Cas9 technology will have on Apicomplexa research. It is expected that genome-wide CRISPR/Cas9 gene knockouts, similar to what has been described for mammals ^115^, will also be published soon for Apicomplexa (preliminary data have been reported already for *T. gondii* ^116^). This will allow researchers to qualify genes as being either essential or not under different *in situ* conditions and to discover so far unknown phenotypes. For instance, parasites deficient in molecules known to target the innate immune system (but also knockdown of host PRRs, especially in primary human cells) will reveal pathways that are necessary for innate sensing and hence parasite control in rodents and humans and help to answer the question of whether actual infection, phagocytosis, or mere contact (“kiss and spit”) is required for CD4 ^+^ and CD8 ^+^ T cell responses.

Moreover, microscopy-based genome-wide screens on CRISPR/Cas9-generated knockouts or fluorescently tagged proteins can be envisaged to follow ^117^. It would allow testing for function and localization of proteins that could not be achieved with this precision, speed, and coverage so far.

In particular, the less-studied stages (e.g. sexual stages within the mosquito [ *Plasmodium*] or the cat [ *T. gondii*]) will be of scientific interest. For the latter, establishing feline organoid cultures that would allow *in vitro* culture of the sexual stages will be crucial ^118^. One of the most eminent questions in this respect is why does sex only take place in cats when this parasite is otherwise so extremely promiscuous in its host range? Together with the advent of modern metabolomics and gene knockouts, the answer to this and other questions might be within reach in the next few years.

Since Apicomplexa are pathogens, the development of new drugs and drug target candidates will of course remain a major driver for studying these parasites, and it is inevitable that there will be a big gain in understanding of the underlying molecular processes. Recent studies on crucial metabolic pathways for lipids ^119, 120^, sugars ^121^, and isoprenoids ^122^ have shown more potential metabolic vulnerabilities of apicomplexan parasites. Moreover, a new class of highly active anti-malarial compounds has already been described ^123, 124^, and one of them, the spiroindolone KAE609, has shown very promising results in recent phase II clinical trials against uncomplicated malaria ^125– 127^. Although drug resistance in the laboratory has already been described for spiroindolones, knowledge about its reported mechanism ^123, 128, 129^ will hopefully help in designing strategies to pre-emptively delay the spread of resistance in nature once KAE609 has been brought onto the market. However, the race is bound to start all over again – winner currently unknown.

## Abbreviations

ACTs, artemisinin combination therapies; BCAA, branched-chain amino acid; ASP5, aspartyl protease 5; BCAT, branched-chain amino acid transferase; BCKDH, branched-chain alpha-ketoacid dehydrogenase; CRISPR, Clustered Regularly Interspaced Short Palindromic Repeats; Cas, CRISPR associated; ETC, electron transport chain; hPINI, human parvulin; IFN-γ, interferon-gamma γ; IFNα/β, interferon α/β; IRG, immunity related GTPases; K13, kelch13; Mavs, mitochondrial antiviral signaling protein; MC, 2-methylcitrate; MYR1, Myc regulation 1; NGS, next-generation sequencing; NOD, Nod-like receptor; PDH, pyruvate dehydrogenase; PEXEL, Plasmodium export element; PI3K, phosphatidylinositol-3-kinase; PI3P, phosphatidylinositol-3-phosphate; PPIase, peptidyl-prolyl isomerase; PRR, pathogen recognition receptor; PTEX, Plasmodium translocon of exported proteins; PVM, parasitophorous vacuolar membrane; ROPs, rhoptry proteins; SEA, South-East Asia; TCA, tricarboxylic acid; *Tg*PFR, *Toxoplasma gondii*-derived protein profilin; TLR, Toll-like receptor.

Dedicated to the memory of Klaus Lingelbach, a devoted *Plasmodium* scientist and a mentor of Frank Seeber.
